# Compounding and Use of Human Medicinal Products in Small Animal Practice: What Are the Perspectives of Veterinarians?—A Pilot Study

**DOI:** 10.3390/vetsci12090914

**Published:** 2025-09-19

**Authors:** Zorana Kovačević, Gordana Gregurić Gračner, Dragana Tomanić, Ksenija Vlahović, Ljubiša Šarić, Dragana Novaković, Ivan Galić, Katarina Pajić, Dragoljub Marić, Marko Samardžija, Olga Sič

**Affiliations:** 1 Department of Veterinary Medicine, Faculty of Agriculture, University of Novi Sad, 21000 Novi Sad, Serbia; 2 Faculty of Veterinary Medicine, University of Zagreb, 10000 Zagreb, Croatia; 3 Institute of Food Technology, University of Novi Sad, 21000 Novi Sad, Serbia; 4 Department of Agricultural Economics and Rural Sociology, Faculty of Agriculture, University of Novi Sad, 21000 Novi Sad, Serbia; 5 Department of Pharmacology and Toxicology, Faculty of Medicine, University of Novi Sad, 21000 Novi Sad, Serbia

**Keywords:** compounding, pets, questionnaire, Serbia, veterinarians

## Abstract

Compounded medications (CMs) have become increasingly relevant in veterinary pharmacotherapy, particularly due to the limited availability of licensed veterinary medicinal products (VMPs). Despite the importance of CMs, data on their use all over the world, as well as in Serbian veterinary practice, remain limited. The aim of this study is to understand Serbian veterinarians’ attitudes towards the use of CMs and human medicinal products in small animal practice, with a focus on challenges and needs they experience related to this type of therapy. A questionnaire-based survey was conducted from January to May 2024 among licensed veterinarians, covering sociodemographics, antibiotic prescribing practices, and attitudes toward CMs. Most respondents (85.4%) reported difficulties in accessing appropriate medications, with 45.8% frequently prescribing human medications for reasons of availability and cost. While 58.3% were familiar with CMs, many emphasized their benefits in tailoring doses and formulations. A majority (65.6%) believed CMs could improve treatment outcomes, yet 87.5% expressed the need for further education. This study shows that Serbian veterinarians frequently prescribe human medications due to limited and costly veterinary medicinal products. While they see the benefits of CMs for personalized treatments, there is a clear need for improved training and clearer regulations to enhance treatment outcomes and ensure safer veterinary care.

## Simple Summary

Veterinarians often face difficulties in finding suitable medications for pets, as only a limited number of products are officially approved for use in animals. To overcome this, they sometimes need to prescribe medications intended for people, or they use specially prepared medications made by pharmacists. To better understand this issue, we conducted a survey among veterinarians in the Autonomous Province of Vojvodina, which is part of the Republic of Serbia. Most veterinarians said that the main problems were the lack of available animal medications and their high cost. The results showed that most veterinarians struggle to obtain the right medications for treatment, and many frequently prescribe human medications because they are more available and affordable. Many were aware of the advantages of specially prepared medications, such as the possibility to adjust the dose or form of the drug to better suit the animal. At the same time, most respondents emphasized the need for more education and clearer rules in this area. These findings have shown that improving access to veterinary medications, together with better training and guidance, could help veterinarians provide safer and more effective treatments for pets.

## 1. Introduction

Compounded medications (CMs) are becoming increasingly important in modern veterinary pharmacotherapy, and it is a field that is constantly evolving. Despite the wide range of therapeutic needs, the availability of licensed medications for different animal species and indications remains limited in the veterinary practice [1]. Recent research indicates that a total of 1564 new medications have been licensed for various animal species [2]. However, this number pales in comparison to the over 35,000 new medications registered for humans, as reported by the U.S. Food and Drug Administration [2]. Hence, to meet the treatment needs of pets, an important aspect of veterinary care is the use of CMs specifically tailored for each species [3].

In Serbia, the treatment of animals is regulated by the Law on Medicines and Medical Devices, which mandates the use of licensed veterinary medicinal products (VMPs). In situations where these products are unavailable, the cascade principle is applied. This principle allows the use of veterinary medications approved for other species or, when necessary, CMs, as long as there are no contraindications [4]. The cascade principle aims to ensure the welfare of animals while maintaining the safety and efficacy of treatments. This regulatory framework aligns with European Union regulations, which also permit the use of approved medications for other species or CMs under certain conditions [5]. However, there may be discrepancies in how these regulations are implemented across different countries, particularly in low-income regions where access to licensed VMPs can be limited. In such cases, CMs and the use of human medicinal products for veterinary purposes may become more prevalent.

It is important to understand the differences in regulations and practices between countries and how these affect the treatment options available to veterinarians, especially in the context of antimicrobial resistance (AMR) and the growing reliance on human medicinal products in veterinary practice. Beyond the limited market of licensed VMPs, their efficacy is further compromised by AMR. To clarify the connection between CMs and AMR, it is important to note that CMs are used when licensed VMPs are unavailable. In veterinary practice, human-approved antibiotics may be prescribed when no suitable veterinary alternatives exist, which has been recognized as a potential driver of AMR in both animals and humans. In such cases, veterinarians may rely on human-approved products or CMs to ensure adequate treatment options for animals. Furthermore, the use of certain antibiotics in veterinary medicine, especially those classified as critically important to human health, raises serious public health concerns due to their potential contribution to the development and spread of AMR [6]. Additionally, a recent study in Serbia examining bacterial isolates from canine otitis revealed significant resistance to several critically important antibiotics used in human medicine, including vancomycin and colistin [7].

In low- and middle-income countries, including Serbia, the lack of commercially available licensed VMPs presents a significant challenge for veterinarians and pet owners, limiting treatment options and affecting both costs and quality of care. One way to address this issue is through the use of CMs and human medicinal products in veterinary practice. However, available data on the use of these therapeutic alternatives in Serbia is limited. Therefore, the aim of this study is to explore Serbian veterinarians’ attitudes towards the use of CMs and human medicinal products in small animal practice, with a particular focus on identifying challenges and needs they face related to this type of therapy.

## 2. Materials and Methods

### 2.1. Ethical Approval

The research was conducted with ethical approval obtained from the Ethics Committee of the Faculty of Agriculture, University of Novi Sad, Serbia (number 1331/1 14 September 2023). Participants were asked to complete a questionnaire following prior instructions from the researchers and after obtaining written informed consent.

### 2.2. Questionnaire Format and Distribution

In this study, the Knowledge, Attitude, and Practice (K—how familiar veterinarians are with CMs and their advantages; A—how veterinarians perceive the use of CMs and human medicinal products in veterinary medicine; P—the actual frequency of prescribing CMs and human antibiotics in veterinary practice) model was applied, which is widely used in research examining.

The survey was created after reviewing the existing, relevant literature on the proposed topic. The questionnaire (Supplementary File S1) was composed of a combination of original questions and questions from various surveys [8,9], with modifications necessary to ensure accurate responses to the questions and statements. The content, comprehension, and readability of the questionnaire were pre-tested on 15 veterinarians from Novi Sad. As a result of the pre-test, some minor modifications were made to the wording of certain questions to enhance clarity and ensure a better understanding of the questions by respondents. Those data were not included in the final analysis. The language of the questionnaire was Serbian, and the translation of certain questions into English was used for clarification during the research process. If necessary, all translations were carefully reviewed to maintain consistency and accuracy in the interpretation of the responses.

The questionnaire was divided into three parts. The first part, entitled “Sociodemographic data”, contained seven questions related to the sociodemographic information of the respondents and some general questions. The second part, entitled “Veterinarians’ prescribing practices particularly regarding human medicinal products and antibiotics”, examined the practice of prescribing human-approved antibiotics. It consisted of three questions about this practice and six additional questions focusing on the factors in decision-making when prescribing antibiotics in their daily work. In the third part of the questionnaire, entitled “Veterinarians’ attitudes and experiences regarding CMs”, the veterinarians’ opinions and practical experiences regarding the use of CMs were explored. It consisted of 11 questions covering general attitudes toward CMs, the frequency of their use, experiences related to obtaining CMs, as well as the challenges veterinarians face when working with CMs.

The questionnaire was then distributed to licensed veterinarians employed in the veterinary clinics located in the territory of the Autonom Province of Vojvodina, South Bačka district, with 350 licensed veterinarians. The research was conducted at the beginning of 2024. Completing the entire questionnaire took about 10 min. The questionnaire was disseminated through the Regional Committee of South Bačka of the Veterinary Chamber of Serbia to licensed veterinarians working in the mixed veterinary practice or the small animal practices. The survey was conducted using the Google Forms platform, with the link sent via email to members of the Veterinary Chamber of Serbia. The survey was available online for 4 months, from 1 January to 1 May 2024. A total of 350 licensed veterinaries were ask to participate; the required sample size was 70 to ensure a valid 95% confidence interval and 2% margin of error if a 20% response rate was obtained. This was then deliberately exceeded to increase the power of the study and to provide for exclusions, dropouts, and the need to perform subgroup analysis. Hence, a total sample size of 96 licensed veterinaries was targeted, with a response rate of 27.43%.

To ensure the reliability and validity of the questionnaire, an internal consistency analysis was conducted using Cronbach’s alpha coefficient. The calculated Cronbach’s alpha was 0.849, indicating the high internal consistency and reliability of the questionnaire. To ensure data entry accuracy, two researchers independently entered the data, and their entries were compared to identify discrepancies. Additionally, the dataset was further analyzed to identify extreme or inconsistent answers that might indicate data entry mistakes, ensuring the reliability and precision of the collected data.

### 2.3. Statistical Analysis

The obtained results were then statistically processed to evaluate the knowledge and attitudes of veterinarians regarding the compounding of medications. The data collected through the survey were analyzed using R software version 4.1.2. Statistical data analysis began with descriptive statistical analysis, followed by Multiple Correspondence Analysis (MCA) providing insights into the relationship between veterinarian’s sociodemographic characteristics and their familiarity with the concept of CMs, and then followed by the application of non-parametric tests to evaluate potential differences among the sociodemographic characteristics of respondents in their attitudes toward the use of CMs. The Mann–Whitney U test and Kruskal–Wallis test were employed for this purpose. The Mann–Whitney U test is used to examine differences between two independent groups on a continuous scale, providing a non-parametric alternative to the parametric *t*-test for independent samples [10]. The Kruskal–Wallis test is used to compare three or more independent groups on a continuous scale [11], offering a non-parametric alternative to one-way analysis of variance (ANOVA). In cases where statistically significant differences were found between groups, the Dunn post hoc test was applied to identify specific groups between which significant differences existed.

## 3. Results

### 3.1. Sociodemographic Characteristics of the Respondents

There were 96 veterinarians included in the study, which included 54 (56.2%) males and 42 (43.8%) females (Table 1).

Respondents were divided into five age groups, with the largest group being 31–40 years (43.8%). Nearly two-thirds (72.9%) work in private institutions, while 27.1% work in public ones. Most respondents hold a Doctor of Veterinary Medicine degree (76.0%), followed by those with a PhD (12.5%), specialist veterinarians (8.3%), and Master’s degree holders (3.1%). Half (50.0%) have been in practice for 6 to 15 years. Furthermore, 60.4% work in a small animal practice, while 39.6% are involved in both large and small animal practices.

### 3.2. Veterinarians’ Prescribing Practices Particularly Regarding Human-Approved Medications and Antibiotics

The majority of respondents (85.4%) answered affirmatively to the question of whether they face challenges regarding the availability of appropriate medications for animals, while only 14.6% answered that they do not encounter this problem. Regarding the frequency of prescribing human-approved medications, 45.8% of respondents indicated that they often prescribe these medications, 40.6% reported that they prescribe them moderately, and 13.5% stated that they rarely or never prescribe human medications.

Respondents identified several reasons for prescribing human medications: 41.7% cited availability, 35.4% pointed to both financial considerations and availability, and 10.4% mentioned financial considerations alone. Other reasons were mentioned by less than 10% (Table 2).

**Figure 1:**
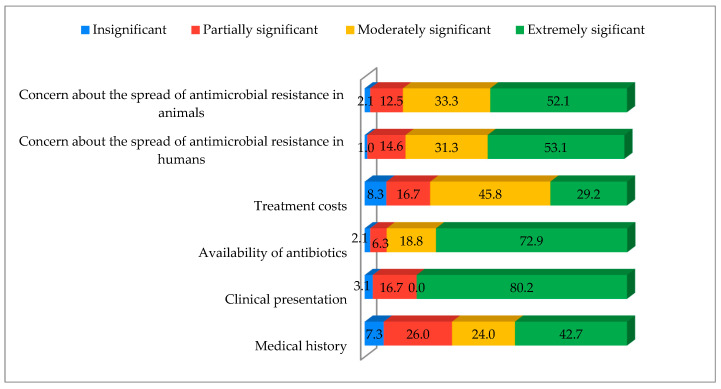
Factors in decision-making when prescribing antibiotics. Factors were rated as follows: 1 = insignificant, 2 = partially significant, 3 = moderately significant, 4 = extremely significant. The numbers in the bars represent the percentages of respondents selecting each option.

Among the factors influencing antibiotic prescription, clinical presentation was considered most important (80.2% of respondents), followed by antibiotic availability (72.9%), AMR in humans and animals (52.1% each), medical history (42.7%), and treatment costs (29.2%) (Figure 1).

### 3.3. Veterinarians’ Attitudes and Experiences Regarding Compounded Medications

Regarding veterinarians’ attitudes and experiences with CMs, 58.3% were familiar with the concept, while the same percentage (58.3%) used CMs moderately. The main advantages noted are dose customization (43.8%), pharmaceutical form customization (27.1%), all listed advantages (19.8%), easier preparation (7.3%), taste customization (1%), and quality control (1%). The vast number of veterinarians (65.6%) believed CMs improve treatment outcomes, whereas 6.3% did not, while 28.1% were uncertain. For obtaining these medications, 66.7% recommend a pharmacy, 26.0% a veterinary clinic, 2.1% mail order services, and 5.2% other options. A significant majority (87.5%) wanted more education on CMs, while only 8.3% believed they are adequately regulated, and 46.9% were uncertain about the current regulatory framework (Table 3).

**Figure 2:**
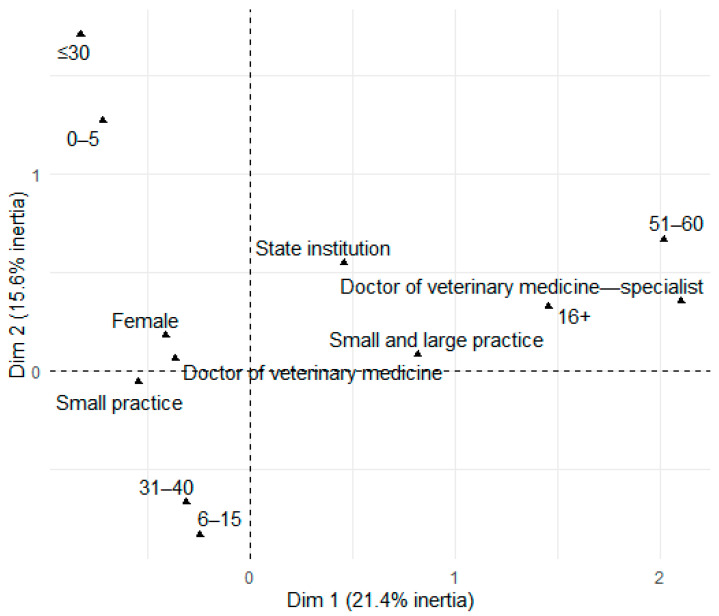
MCA biplot: Sociodemographics and familiarity with compounded medications.

Based on the MCA biplot (Figure 2), several key patterns were identified, indicating different groups of respondents in relation to their familiarity with CMs. Firstly, it was observed that respondents who indicated that they had educated themselves independently about CMs were predominantly male and belonged to younger age groups, particularly those under 30 years of age.

**Figure 3:**
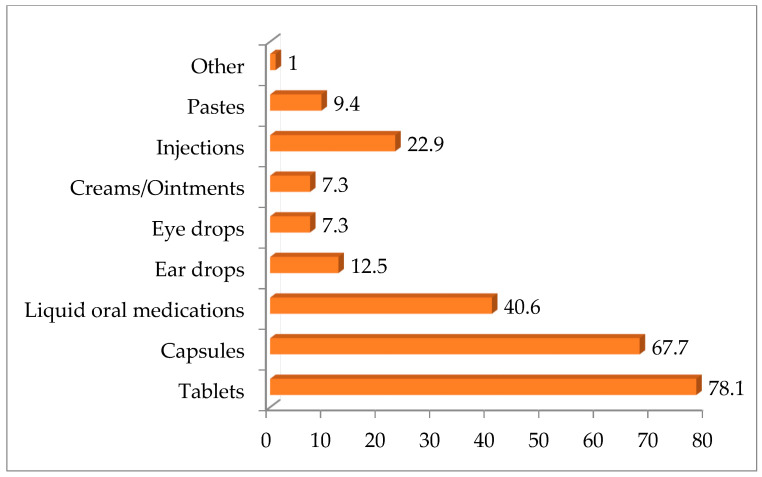
Types of pharmaceutical dosage forms of VMPs that often require dose or form adjustment. The numbers in the bars represent the percentages of respondents selecting each option.

Participants were surveyed on which veterinary medication formulations frequently require dose or formulation adjustments (Figure 3). The majority reported tablets (78.1%) and capsules (67.7%) as requiring adjustment, followed by oral liquids (40.6%) and injections (22.9%). Creams/ointments and eye drops were less commonly mentioned, each by 7.3% of respondents.

**Figure 4:**
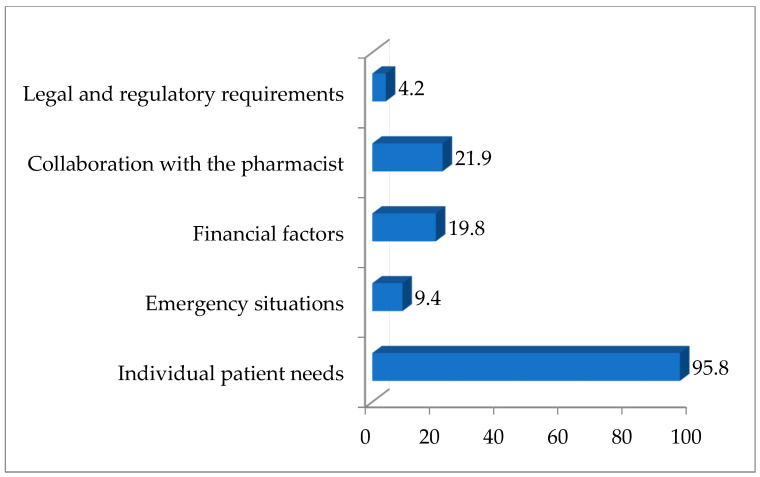
The percentage of respondents selecting certain factor influencing the decision to use compounded medications instead of commercially available medications.

Respondents identified factors influencing their choice to use CMs over commercially available ones (Figure 4). The most cited reason was individual patient needs (95.8%), followed by collaboration with a pharmacist (21.9%) and financial factors (19.8%). Emergency situations (9.4%) and legal/regulatory considerations (4.2%) were less frequently mentioned.

**Figure 5:**
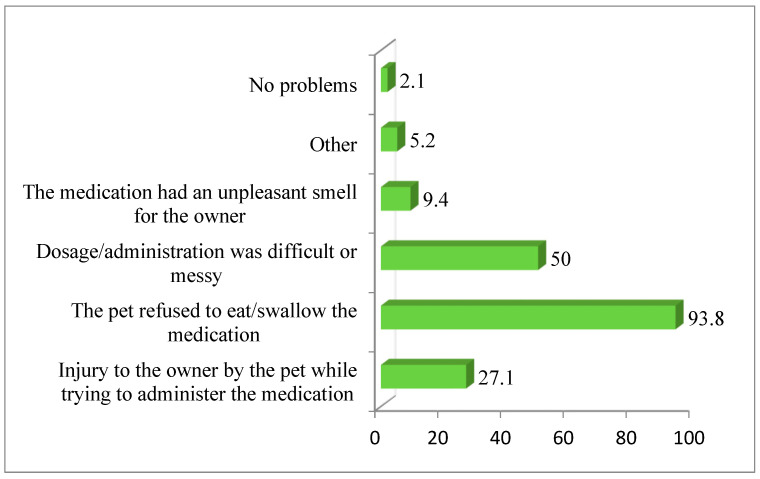
The percentage of respondents selecting certain options due to the biggest problems pet owners face when administering medications to their pets.

The main problems reported when administering medication to pets included refusal to eat or swallow (93.8%), difficulty with dosage or application (50%), and risk of owner injury (27.1%). Less commonly reported issues were unpleasant odor (9.4%), other problems (5.2%), and no difficulties (2.1%) (Figure 5).

**Figure 6:**
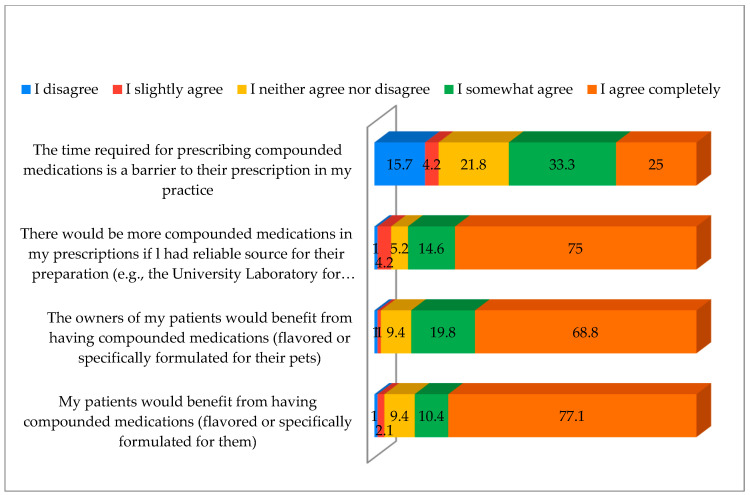
Participants’ attitudes towards the use of compounded medications. Factors were rated as follows: 1 = disagree, 2 = slightly disagree, 3 = neutral, 4 = somewhat agree, 5 = agree completely. The numbers in the bars represent the percentages of respondents selecting each option.

Figure 6 illustrates respondents’ attitudes towards CMs, measured on a Likert scale from 1 (strongly disagree) to 5 (agree completely). It can be observed that respondents largely agreed with the statement that their patients would benefit from having medications compounded (with flavor or formulated specifically for them), as well as with the idea that they would prescribe more CMs if they had a reliable source for their preparation. The least agreement was expressed towards the statement that the time required for prescribing CMs is a barrier to their use in the practice.

A statistically significant difference was observed in gender among respondents only in their attitude towards whether there would be more CMs in their prescriptions if they had a reliable source for their preparation (*p* = 0.029), with males showing a higher degree of agreement with this statement (Table 4). For all other statements, no statistically significant differences were found by gender (*p* > 0.05). Statistical analysis revealed significant differences between the age groups of respondents and their opinion on the availability of reliable sources and their production (*p* = 0.026), while the post hoc test revealed that there are significant differences among younger respondents (up to 30 years old) and those aged 51–60 years (*p* = 0.026) and older than 60 years (*p* = 0.021); younger respondents consider this statement more significant. No other age-related differences were significant (*p* > 0.05). Employment institution impacted the belief that the time required for prescribing CMs is a barrier, with state institution employees showing less agreement compared to private institution employees (*p* = 0.048). Attitudes also significantly varied by years of practice and the availability of CMs (*p* = 0.035); post hoc test results showed significant differences between those with up to 5 years compared to those with 6–15 years of practice (*p* = 0.048). Small practice respondents showed higher agreement on prescribing more CMs with a reliable source (*p* = 0.012). A statistically significant difference in the number of cases per month was found for the statement that the owners will benefit from CMs (*p* = 0.041). Post hoc test results showed that there is a statistically significant difference between those with over 300 patients monthly and all other categories (*p* < 0.05); those with over 300 patients monthly believe that the time required for prescribing compounded medication is not a barrier in their practice (Table 4).

## 4. Discussion

To the best of our knowledge, only one study has explored veterinarians’ knowledge and attitudes toward CMs [8], while none addressed the prescribing of human medications. Such research is important given the limited availability of VMPs in Serbia and other low-income countries. Furthermore, a study from Brazil showed that veterinarians increasingly prescribe human-approved antibiotics for companion animals, especially penicillin, cephalosporins, and azithromycin, with significant regional disparities, underscoring the need for better regulation and stewardship [12]. In addition, a study from Thailand examined antibiotic use in dogs and cats at veterinary teaching hospitals, reporting high consumption of critically important antimicrobials for humans and highlighting the need for rational prescribing and monitoring [13].

The lengthy registration process often limits the supply of licensed VMPs [14], with 85.4% of veterinarians reporting availability issues in our study. As a result, veterinarians adapt licensed medications through the prescribing cascade, using alternatives when suitable VMPs are unavailable, thereby ensuring animal welfare, despite regulatory and supply challenges [14,15,16]. Given these limitations, it is not surprising that many veterinarians turn to human medicinal products as an alternative. Actually, our study shows that 45.8% of veterinarians frequently prescribe human medications. The main reasons reported by veterinarians in our study were availability (41.7%), a combination of cost and availability (35.4%), and cost alone (10.4%). Within this group of human medicinal products, antibiotics are of particular relevance due to their frequent use in veterinary practice and their potential impact on AMR. According to the European Medicines Agency (EMA) [17], the off-label use of antibiotics in dogs and cats ranges from 13 to 80%, depending on regulations, availability, and survey periods [18,19,20]. These findings align with Hölsö et al. [18], who linked frequent prescription of human antibiotics to the lack of suitable veterinary options. In some cases, human antibiotics are chosen for their lower cost, even when veterinary medications are available [21,22], or because they are not subject to withdrawal periods in pets [18]. EMA also notes that newer generations of human medicinal products are sometimes used in small animals without clear justification [17].

AMR is a major global health threat, driven by irrational antibiotic use in veterinary medicine [23]. To protect antibiotics critical for humans, the World Health Organization recommends limiting their veterinary use [24]. Our study highlights concerning practices reported in the literature, including the frequent use of critically important antibiotics in companion animals, sometimes without prior susceptibility testing [25], alongside evidence from Serbia showing non-compliance with prudent-use guidelines [26]. Beyond reducing use of critically important antibiotics [24], rational use of existing ones is essential, which can be supported by compounding to optimize dosage and minimize misuse. Identification of the factors influencing antibiotic prescribing is key to improving use in veterinary medicine and slowing drug resistance [27]. Our results show that veterinarians prioritized clinical presentation (80.2%), medical history (42.7%), and antibiotic availability (72.9%) over treatment costs and AMR concerns (52%). These findings align with Servia-Dopazo et al. [27] and previous studies highlighting the importance of availability [9,28,29,30,31] and treatment costs [30,32,33].

The growing presence of specialized veterinary compounding pharmacies highlights the widespread use of this practice, particularly in the United States, where over 12,000 compounding pharmacies exist, with roughly a quarter of their products intended for animals [3,14,34,35]. Australia also has specialized veterinary pharmacies, though their exact number is unclear, and in New Zealand, one pharmacy offers compounding services [35]. The U.S. Food and Drug Administration estimates that 75,000 pharmacies prepare 6,350,000 compounded animal prescriptions annually [36]. While European data are scarce, a survey in the Czech Republic and Slovakia found that veterinarians typically prescribe no more than one CM per day [37]. Actually, access to compounding pharmacy services supports safe and effective treatment, with CMs gaining popularity among veterinarians, owners, and breeders [14]. This is reflected in the current study, where over half of the veterinarians (58.3%) were familiar with CMs. MCA analysis showed that younger veterinarians often learn about CMs independently, while familiarity was higher among those with 6–15 years of experience working in private practice. In contrast, limited knowledge was associated with respondents from state institutions and with less continuing professional education, highlighting the roles of professional development and the workplace environment in shaping exposure to CMs.

CMs provide benefits such as dose adjustment, modified forms, and easier administration [38]. In our study, 43.8% of respondents cited dose adjustment, 27.1% form modification, and 7.3% easier administration, while 65.6% believed CMs improve treatment outcomes. Most recommended obtaining CMs from pharmacies (66.7%) or veterinary clinics (26.0%). Simple preparations can be made by veterinarians, but complex ones require pharmacists, underscoring the need for collaboration between the two professions [1,14,38,39].

Greater education and communication between veterinarians and pharmacists are essential for patient care [1]. Most respondents (87.5%) wanted more education on CMs, especially younger veterinarians and those in state institutions, highlighting the need for targeted professional development. Only 8.3% believed CMs are adequately regulated, while nearly half were unsure. Although the need for CMs is well established, regulatory support is limited due to safety risks and counterfeit VMPs [14]. In Serbia, compounding is regulated by the Medicines and Medical Devices Act under the Ministry of Health, with pharmacies required to follow Good Pharmacy Practice standards but not GMP [1]. Veterinary pharmacies producing these medications must have proper facilities and equipment for preparation and quality control [4].

Tablets are the most common oral formulation for pets, offering stability and precise dosing, though bioavailability varies by species. To improve suitability and palatability, tablets are often crushed, capsules reformulated, or solutions adjusted [39,40]. The most frequent forms that are adjusted are tablets (78.1%) and capsules (67.7%), followed by oral liquid medications (40.6%) and injections (22.9%). Individual patient needs were cited by the majority of respondents (95.8%) as the main reason for using CMs, reflecting the trend toward personalized veterinary therapy [3]. This practice allows veterinarians to effectively treat different animal species, taking into account their unique characteristics, as well as any specific requirements or limitations [41,42]. Less frequently reported factors included collaboration with a pharmacist (21.9%), financial considerations (19.8%), and emergency situations (9.4%), while regulatory aspects were rarely mentioned (4.2%), indicating limited awareness and country-specific legislative differences [1].

In veterinary practice, owners face challenges with pet medication compliance, especially with tablets. Pets may refuse to consume the entire tablet or take only a partial dose, which is particularly problematic with cats [39,40]. Survey results reflect veterinarians’ perspectives on pet owners’ challenges, showing that the main issues reported were pets refusing medication (93.8% of respondents), difficulties with dosing/administration (50%), and injury to the owner during medication attempts (27.1%).

There are several notable limitations in the study. Firstly, although our respondents’ demographic characteristics were similar to those of the Serbian veterinarian population, the small sample size and non-response bias mean the results of this study should be interpreted with caution. These findings may not represent all small animal veterinarians in Serbia and could differ and be less applicable in other regions of country. Besides regional differences, variations in practice size and levels of experience may influence the responses and thus limit the generalizability of the results. Secondly, the study relies on self-reported data from veterinary practitioners, which may be subject to reporting bias or inaccuracies. Veterinarians might overstate their knowledge of AMR or the use of CMs due to social desirability or a lack of detailed records. Additionally, the study focuses primarily on human medicinal products and systemic antibiotics, which may not capture the full spectrum of medication use or the complexity of prescribing practices. This narrow focus might overlook other important aspects of veterinary pharmacotherapy and its impact on AMR. Finally, the study does not account for potential confounding factors, such as differences in practice guidelines, regional regulations, or the availability of CMs, which could affect the results. Further research is needed to address these limitations and provide a more comprehensive understanding of the use of human medicinal products, antibiotics, and compounded treatments in veterinary practice.

## 5. Conclusions

This study highlights the challenges Serbian veterinarians face regarding the limited availability of VMPs, which often leads to the frequent prescribing of human medications and the use of CMs. While clinical presentation and availability remain the most important drivers of antibiotic prescribing, concerns about AMR were not always prioritized, underlining the need for more rational prescribing practices. The findings also emphasize the growing role and acceptance of CMs, especially for dose and form adjustments to improve patient compliance and treatment outcomes. However, gaps in knowledge, limited regulatory oversight, and a strong demand for additional education point to areas where professional development and policy support are urgently needed. Further research is needed to explore the long-term impact of these practices on AMR, the effectiveness of educational initiatives, and the potential benefits of enhanced collaborations between veterinarians and pharmacists.

## Figures and Tables

**Table 1:** Sociodemographic characteristics of the respondents.

Variable	Response	*n*	%
Gender	Female	42	43.8
	Male	54	56.2
Age group	≤30	18	18.8
	31–40	42	43.8
	41–50	22	22.9
	51–60	12	12.5
	61+	2	2.1
Work sector	State institution Private institution	26	27.1
		70	72.9
Level of education	Doctor of veterinary medicine Master of veterinary medicine Doctor of medical sciences—veterinary medicine Doctor of veterinary medicine—specialist	73	76
		3	3.1
		12	12.5
	
		8	8.3
Number of years working in practice	0–5	26	27.1
	6–15	48	50
	16+	22	22.9
Type of practice	Small practice	58	60.4
	Small and large practice	38	39.6
Number of monthly cases	0–100	50	52.1
	101–200	21	21.9
	201–300	15	15.6
	301+	10	10.4

**Table 2:** Descriptive statistics of questions on medication prescribing practices.

Variable	Response	*n*	%
Do you face challenges regarding the availability of appropriate medications for animals?	Yes	82	85.4
	No	14	14.6
How often do you prescribe human medicinal products?	Often	44	45.8
	Moderately	39	40.6
	Rarely or never	13	13.5
What are the reasons for prescribing human medicinal products?	Financial situation of pet owners	10	10.4
	Prescribing practices	0	0
	Availability of medication	40	41.7
	Pressure from owners	1	1
	The financial situation of pet owners and the availability of medications	34	35.4
	The financial situation of pet owners, the availability of medications, and prescribing practices	6	6.3
	The financial situation of pet owners, the availability of medications, and pressure from owners	3	3.1
	Prescribing practices and the availability of medications	2	2.1

**Table 3:** Descriptive statistics of veterinarians’ attitudes and experiences regarding compounded medications.

Variable	Response	*n*	%
Are you familiar with the concept of compounding medications?	Yes	56	58.3
	No	21	21.9
	I have educated myself independently	19	19.8
How often do you use compounded medications in your daily practice?	Often	0	0
	Moderately	56	58.3
	Never	40	41.7
What do you consider to be the main advantages of compounded medications?	Dose adjustment for the patient	42	43.8
	Customization of the pharmaceutical dosage form for the patient	26	27.1
	Customization of the taste for the patient	1	1
	Controlled quality	1	1
	Easier preparation	7	7.3
	Fast delivery	0	0
	All of the above	19	19.8
Do you think that compounded medications contribute to better treatment outcomes for certain patients?	Yes	63	65.6
	No	6	6.3
	Unsure	27	28.1
Where would you recommend pet owners procure compounded medications for their pets?	Veterinary clinic	25	26
	Pharmacy	64	66.7
	By post office	2	2.1
	Other	5	5.2
Would you like more education about compounded medications to better understand their capabilities and limitations?	Yes	84	87.5
	No	4	4.2
	Unsure	8	8.3
Do you think the compounding of veterinary medications is adequately regulated in your field of work?	Yes	8	8.3
	No	43	44.8
	Unsure	45	46.9

**Table 4:** Respondents’ attitudes toward the use of compounded medication in relation to respondents’ gender, age, work sector, level of education, number of years working in practice, type of practice, and number of monthly cases.

		My Patients Would Benefit from Having Their Medications Compounded (with Flavoring or Formulated Specifically for Them)	The Owners of My Patients Would Benefit from Having Their Medications Compounded (with Flavoring or Formulated Specifically for Their Pet)	There Would Be More Compounded Medications in My Prescriptions If I Had a Reliable Source for Their Preparation (e.g., a University Compounding Laboratory in the Department of Veterinary Medicine)	The Time Required to Prescribe Compounded Medications Is a Barrier to Their Prescription in My Practice
		$\overset{-}{x}$ ± SD	$\overset{-}{x}$ ± SD	$\overset{-}{x}$ ± SD	$\overset{-}{x}$ ± SD
Gender	Male Female	4.762 ± 0.753	4.690 ± 0.795	4.854 ± 0.905	3.341 ± 1.285
		4.596 ± 0.697	4.538 ± 0.517	4.538 ± 0.786	3.500 ± 1.356
		U = 962.000; *p* = 0.166	U = 1028.0000; *p* = 0.544	U = 860.000; *p* = 0.029	U = 1133.000; *p* = 0.591
Age group	≤30 31–40 41–50 51–60 61+	4.333 ± 0.970	4.500 ± 0.786	5.000 ± 0.010	3.167 ± 1.543
		4.762 ± 0.670	4.714 ± 0.564	4.643 ± 0.905	3.643 ± 1.224
		4.905 ± 0.338	4.762 ± 0.542	4.667 ± 0.961	3.619 ± 1.358
		4.455 ± 0.535	4.091 ± 0.900	4.300 ± 0.756	2.900 ± 0.900
		4.500 ± 0.957	4.500 ± 1.000	4.000 ± 1.069	2.000 ± 0.787
		H = 9.071; *p* = 0.059	H = 6.687; *p* = 0.153	H = 11.083; *p* = 0.026	H = 7.963; *p* = 0.093
Work sector	State institution Private institution	4.640 ± 0.700	4.560 ± 0.860	4.760 ± 0.905	3.000 ± 1.356
		4.681 ± 0.071	4.623 ± 0.623	4.632 ± 0.843	3.588 ± 1.269
		U = 893.000; *p* = 0.715	U = 837.500; *p* = 0.790	U = 845.500; *p* = 0.957	U = 1261.000; *p* = 0.048
Level of education	Doctor of veterinary medicine Master of veterinary medicine Doctor of medical sciences—veterinary medicine Doctor of veterinary medicine—specialist	4.667 ± 0.736	5.000 ± 0.637	4.818 ± 0.788	4.375 ± 1.385
		4.639 ± 0.000	4.667 ± 0.577	4.818 ± 0.577	4.000 ± 0.577
		4.690 ± 0.603	4.667 ± 0.788	4.818 ± 1.267	4.250 ± 1.379
		3.479 ± 0.518	3.667 ± 0.926	3.091 ± 0.707	3.375 ± 0.518
		H = 7.238; *p* = 0.065	H = 7.151; *p* = 0.067	H = 7.693; *p* = 0.053	H = 1.579; *p* = 0.664
Number of years working in practice	0–5 6–15 16+	4.538 ± 0.859	4.754 ± 0.706	4.667 ± 0.392	4.670 ± 1.419
		4.538 ± 0.642	4.702 ± 0.595	4.476 ± 0.986	4.606 ± 1.320
		4923 ± 0.587	4.574 ± 0.854	4.550 ± 0.854	4.667 ± 1.167
		H = 1.142; *p* = 0.565	H = 1.283; *p* = 0.526	H = 6.713; *p* = 0.035	H = 7.963; *p* = 0.093
Type of practice	Small practice Small and large practice	4.649 ± 0.767	4.684 ± 0.602	4.807 ± 0.755	3.632 ± 1.175
		4.703 ± 0.577	4.486 ± 0.760	4.444 ± 0.942	3.111 ± 1.467
		U = 1027.500; *p* = 0.770	U = 907,500; *p* = 0.157	U = 793,500; *p* = 0.012	U = 844.000; *p* = 0.137
Number of monthly cases	0–100 101–200 201–300 301+	4.551 ± 0.739	4.857 ± 0.710	4.714 ± 0.819	4.670 ± 1.384
		4.429 ± 0.529	4.810 ± 0.332	4.786 ± 0.529	4.606 ± 1.173
		4.646 ± 0.816	4.857 ± 0.907	4.571 ± 1.231	4.667 ± 1.227
		3.497 ± 0.647	2.952 ± 0.647	3.500 ± 0.647	3.430 ± 0.674
		H = 3.722; *p* = 0.293	H = 8.282; *p* = 0.041	H = 2.649; *p* = 0.449	H = 6.746; *p* = 0.080

Values are presented as mean ± SD (Likert 1–5). For two-level factors (Gender, Work sector, Type of practice), groups were compared with the Mann–Whitney U test; for multi-level factors (Age group, Level of education, Years in practice, Number of monthly cases), with the Kruskal–Wallis H test. Reported *p*-values refer to overall between-group comparisons; Dunn’s post hoc tests were performed where applicable and are described in the text. $\overset{-}{x}$ = mean; SD = standard deviation; U = Mann–Whitney U test; H = Kruskal–Wallis H test; *p* = *p*-value.

## Data Availability

The original contributions presented in this study are included in the article/Supplementary Material. Further inquiries can be directed to the corresponding author.

## Supplementary Materials

The following supporting information can be downloaded at: https://www.mdpi.com/article/10.3390/vetsci12090914/s1, Supplementary File S1: Questionnaire.

## Abbreviations

The following abbreviations are used in this manuscript:

CMs	Compounded medications
VMPs	Veterinary medicinal products
AMR	Antimicrobial resistance
