# Supplementary material for: Compounding and Use of Human Medicinal Products in Small Animal Practice: What Are the Perspectives of Veterinarians?—A Pilot Study
Source: Vet Sci. 2025 Sep 19;12(9):914. doi: 10.3390/vetsci12090914 (PMC12474484; doi:10.3390/vetsci12090914)
Supplement: Supplementary file 1 [file vetsci-12-00914-s001.zip › vetsci-3867434-supplementary.pdf]

Compounding and Use of Human Medicinal Products in Small Animal Practice: What Are the Perspectives of Veterinarians? – A Pilot Study

I Sociodemographic data

1. Gender (circle):
   a) female
   b) male

2. Year of birth (write) ___________

3. Do you work in (circle):
   a) a public institution
   b) a private institution

4. Your highest level of education (circle):
   a) Doctor of Veterinary Medicine
   b) Master of Veterinary Medicine
   c) Doctor of Medical Sciences - Veterinary Medicine
   d) Doctor of Veterinary Medicine - Specialist

5. Number of years you worked in practice (circle):
   a) 0 - 5
   b) 6 - 15
   c) > 15

6. Type of practice (circle):
   a) small animal practice
   b) small and large animal practice

7. How many patients do you examine on average per month? (write): ______

II Veterinarians' prescribing practices particularly regarding human medicinal products and antibiotics

1. Do you face challenges regarding the availability of appropriate medications for animals? (circle):

yes/no

2. How often do you prescribe human medicinal products? (circle):

often / moderately / rarely or never

3. What are the reasons for prescribing human medicinal products? (Multiple answers):

a) Financial situation of the animal owner

b) Prescription practices

c) Medication availability

d) Pressure from the animal owner

4. The significance of factors in deciding when prescribing antibiotics:

Anamnesis	Extremely significant / Moderately significant / Partially significant / Insignificant
Clinical presentation	Extremely significant / Moderately significant / Partially significant / Insignificant
Availability of antibiotics	Extremely significant / Moderately significant / Partially significant / Insignificant
Treatment costs	Extremely significant / Moderately significant / Partially significant / Insignificant
Concern about the spread of antimicrobial resistance in humans	Extremely significant / Moderately significant / Partially significant / Insignificant
Concern about the spread of antimicrobial resistance in animals	Extremely significant / Moderately significant / Partially significant / Insignificant

III Veterinarians' attitudes and experiences regarding compounded medications

1. Are you familiar with the concept of compounding medications? (circle):

a) yes

b) no

c) I educated myself independently about compounded medications using available scientific sources of information.

2. How often do you use compounded medications in your daily practice? (circle):

often / moderately / never

3. What do you consider to be the main advantages of compounded medications?

a) Dose adjustment for the patient
b) Customization of the pharmaceutical dosage form for the patient
c) Customization of the taste to the patient
d) Controlled quality
e) Easier preparation
f) Fast delivery
g) All of the above

4. Do you think that compounded medications contribute to better treatment outcomes for certain patients? (circle):

yes / no / Unsure

5. Where would you recommend pet owners to procure compounded medications for their pets? (circle):

a) veterinary clinic
b) pharmacy
c) by post-office
d) other

6. Would you like more education about compounded medications to better understand their capabilities and limitations? (circle):

yes / no / Unsure

7. Do you think the compounding of veterinary medications is adequately regulated in your field of work? (circle):

yes / no / Unsure

8. Which types of pharmaceutical forms of veterinary drugs often require dose or form adjustments? (Multiple answers):

a) tablets
b) capsules
c) liquid oral medications (solutions, suspensions)
d) ear drops
e) eye drops
f) creams/ointments
g) injections
h) pastes
i) other (please specify___________)

9. What factors influence your decision to use compounded medications instead of commercially available, manufactured drugs? (Multiple answers):

a) Individual patient needs
b) Emergency situations
c) Financial factors
d) Collaboration with the pharmacist
e) Legal and regulatory requirements

10. From your experience, what are the main challenges pet owners face when administering medication to their pets? Please check all that apply (Multiple answers):

a) Injury to the owner caused by the pet while trying to administer the medication
b) Pet refused to eat/swallow the medication
c) Dosage/administration of the medication was difficult or messy
d) Medication had an unpleasant odor for the owner
e) Other
f) No problems

11. On the scale provided next to each statement, indicate the extent to which you agree with it. One response per statement.

		I completely agree	I somewhat agree	Neither agree nor disagree	Slightly agree	Disagree
1.	My patients would benefit from having compounded medications (flavored or specifically formulated for them)	5	4	3	2	1
2.	The owners of my patients would benefit from having compounded medications (flavored or specifically formulated for their pets)	5	4	3	2	1
3.	There would be more compounded medications in my prescriptions if I had a reliable source for their preparation (e.g., the University Laboratory for Compounded Medications at the Department of Veterinary Medicine)	5	4	3	2	1
4.	The time required for prescribing compounded medications is a barrier to their prescription in my practice	5	4	3	2	1
